# Evaluation and Prediction of Post-stroke Cerebral Edema Based on Neuroimaging

**DOI:** 10.3389/fneur.2021.763018

**Published:** 2022-01-11

**Authors:** Xiaocheng Zhang, Peiyu Huang, Ruiting Zhang

**Affiliations:** Department of Radiology, School of Medicine, The Second Affiliated Hospital of Zhejiang University, Hangzhou, China

**Keywords:** ischemic stroke, edema, mechanism, imaging evaluation, predictors

## Abstract

Cerebral edema is a common complication of acute ischemic stroke that leads to poorer functional outcomes and substantially increases the mortality rate. Given that its negative effects can be reduced by more intensive monitoring and evidence-based interventions, the early identification of patients with a high risk of severe edema is crucial. Neuroimaging is essential for the assessment and prediction of edema. Simple markers, such as midline shift and hypodensity volume on computed tomography, have been used to evaluate edema in clinical trials; however, advanced techniques can be applied to examine the underlying mechanisms. In this study, we aimed to review current imaging tools in the assessment and prediction of cerebral edema to provide guidance for using these methods in clinical practice.

## Introduction

Cerebral edema is a severe complication of acute ischemic stroke; it results in poorer functional outcomes and significantly increases the rate of mortality. During ischemia, excessive fluid accumulates in the intracellular or extracellular spaces of the brain because of the failure of energy-dependent ion transport (1–3) and the destruction of the blood–brain barrier (BBB) (4–6), which leads to tissue swelling and the elevation of intracranial pressure. Cerebral edema can appear several hours after ischemia and may progress over the first few days of stroke onset (7).

Patients with mild to moderate edema can be treated with medication (3, 8, 9). However, for those with malignant edema (ME), the mortality rate can increase up to 80% with conservative treatment (10–12). Timely surgical treatment by early decompressive craniectomy is recommended to reduce mortality (9, 13–15). Thus, the accurate assessment and early prediction of edema can benefit patients by more intensive monitoring and evidence-based interventions.

Neuroimaging examination is essential for the assessment of edema. Midline shift (MLS) has long been established as a marker of severe edema and is known to correlate with clinical deterioration (16–18). However, MLS is insensitive to mild to moderate edema and is thus an unsuitable index for monitoring the condition of patients. Recently, new methods, such as cerebrospinal fluid (CSF) displacement (19, 20) and net water uptake (NWU) (12, 21), have been developed to quantify brain edema at an early stage. Furthermore, magnetic resonance imaging (MRI) can distinguish cytotoxic edema from ionic edema and vasogenic edema (22, 23), and advanced MRI techniques, such as diffusion tensor imaging, can detect subtle structural changes in brain edema (24). These new methods can be used to quantify less severe edema and may serve as early imaging markers in clinical practice.

Imaging is important in the prediction of edema. Previous research has shown that infarct volume, collateral status, and CSF volume (CSV), among other factors, are closely related to the development of edema (11, 18, 25, 26). However, the predictive values of these indicators often vary across studies (18, 27); moreover, the optimal timing and modality of imaging exams are also under debate.

There have been numerous reviews on post-stroke edema, of which most have focused on the mechanisms of edema with an emphasis on scientific discoveries rather than on clinical applications. In this review, we briefly discuss the mechanisms of post-stroke edema and focus more on current edema assessment methods and neuroimaging predictors.

Our aim was to provide a comprehensive review of imaging tools for the management of edema and provide guidance for clinical practice.

## Mechanism of Edema

Traditionally, cerebral edema after an ischemic stroke includes cytotoxic edema, ionic edema, and vasogenic edema (7). These three processes occur in sequence and are closely interrelated. Cytotoxic edema is the earliest manifestation of brain hydromineral disturbance as a result of changes in ion channel and transporter (e.g., Na^+^-K^+^-2Cl^−^ cotransporter and Na-H exchangers) activity during a stroke event (3, 28–31). It attracts ions and water into neurons or astrocytes, which causes intracellular water accumulation and extracellular space reduction without increasing brain tissue volume. Because of the changes in ion concentrations on both sides of the BBB, new gradients are formed; this generates a driving force for an influx of water and ion across the intact BBB into the depleted extracellular space, resulting in ionic edema (32–34). Ischemia can also activate inflammatory mediators and increase oxidative stress (35, 36), both of which lead to BBB disruption. This event allows plasma proteins and other macromolecules to pass through the BBB from the intravascular space into brain tissue, which further aggravates water influx and results in vasogenic edema. Recently, studies have shown that post-stroke cerebral edema can also be driven by CSF influx through perivascular spaces (37, 38). Although this has not been confirmed clinically, the related mechanisms underlying the structural and functional abnormalities of the brain's glymphatic system following stroke have gathered the interest of researchers.

## Imaging Evaluation of Cerebral Edema

### Computed Tomography

Computed tomography (CT) is the most frequently used diagnostic procedure in acute stroke. It is sensitive to net water changes but not fluid shifts among tissue compartments within the brain parenchyma. Therefore, CT is sensitive to ionic and vasogenic edema but not cytotoxic edema because fluid shifts from the vascular to interstitial spaces during the first two processes (22, 39, 40). Experimental models have demonstrated a linear relationship between CT attenuation and hemispheric tissue water content (39–41). Within the first few hours following stroke onset, CT shows attenuation of gray matter, which results in the loss of gray/white matter contrast in the cortex, indistinct basal ganglia, and an insular ribbon. With the development of edema, cortical sulci may disappear, and hypoattenuation develops in white matter. During the late stages, ventricles may shrink because of increased parenchymal volume, and MLS occurs.

#### Midline Shift and Volume of Infarct-Related Hypodensity

MLS and the volume of infarct-related hypodensity are two commonly used imaging markers of edema based on CT images (16–18). MLS describes the degree of displacement of the septum pellucidum, which is a thin membrane between the frontal horns of the lateral ventricles, relative to the ideal midline on CT images. An MLS of over 5 mm is usually considered malignant cerebral edema (18, 42). Edema in a temporal lobe infarction can cause uncal herniation with severe symptoms despite minimal MLS.

The volume of infarct-related hypodensity has also been widely used to evaluate edema (22, 25), and it can be manually outlined on each CT slice. However, quantifying hypodensity on CT during the acute phase when infarcts are subtle can sometimes be difficult; moreover, distinguishing edema from infarct growth in follow-up studies is also challenging.

**Figure 1:**
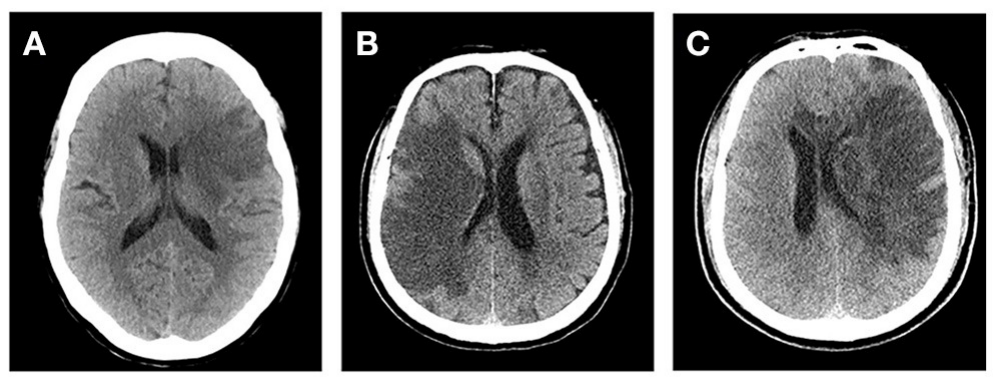
Cerebral edema grades. **(A)** Left frontal lobe and basal ganglia brain swelling, <1/3 of the hemisphere. **(B)** Right frontal and parietal lobe brain swelling, >1/3 of the hemisphere and without midline shift (MLS). **(C)** Left cerebral hemisphere swelling, >1/3 of the hemisphere with MLS.

At present, several comprehensive scores are based on the above methods, such as cerebral edema grading (grade 1 = focal brain swelling of ≤ 1/3 of the hemisphere, grade 2 = >1/3 of the hemisphere, grade 3 = edema with MLS; Figure 1). These scores are used widely in clinical studies (43–45). However, this semi-quantitative method can only be applied to roughly assess the degree of edema, and it is insufficient for evaluating patients with mild edema.

#### Displacement of Cerebrospinal Fluid

**Figure 2:**
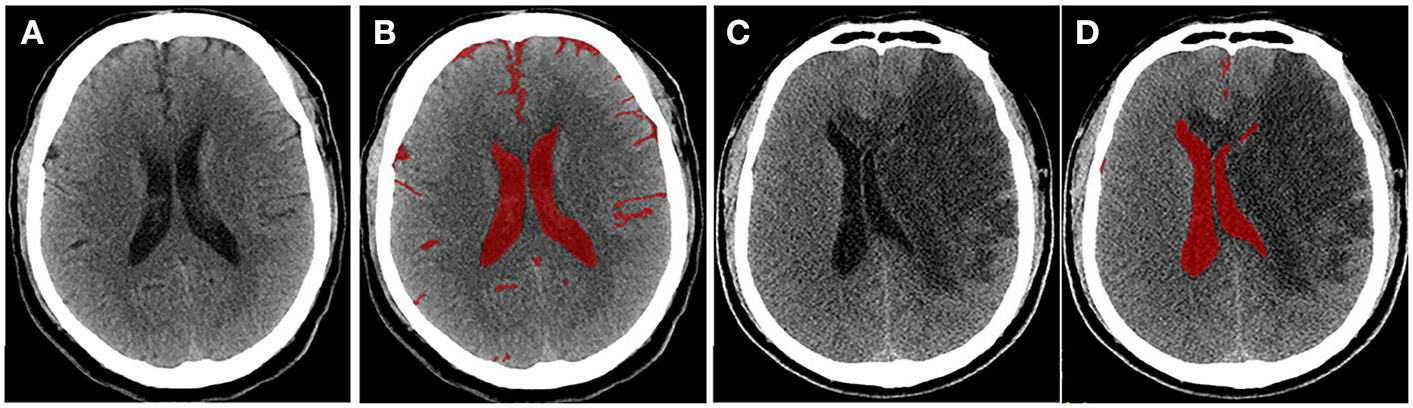
Quantitative cerebrospinal fluid (CSF) shifts. Baseline **(A)** and 24 h **(C)** follow-up CTs after ischemic stroke, respectively. CSF spaces are outlined on a CT scan slice at baseline **(B)** and follow-up **(D)**. Supratentorial CSF and basal cisterns were outlined on each slice, and ΔCSF was subsequently calculated.

To quantify the severity of edema more accurately, Dhar et al. proposed a CT-based volumetric measure of CSF shifts over time (19). As ionic and vasogenic edemas develop, the CSF is progressively displaced from the sulci and ventricles of the cerebral hemispheres to compensate for the increased brain tissue volume in the fixed cranial cavity. Supratentorial CSF spaces (sulci and ventricles ipsilateral and contralateral to the stroke and the third ventricle) and basal cisterns are outlined on each slice, and the volume of CSF is quantified (Figure 2). The CSF can be pushed out of the hemispheric sulci, cerebral ventricles, and basal cisterns as edema develops in the hours or days following the stroke. Studies have shown that, compared with MLS, the reduction in CSV (ΔCSF) from baseline to follow-up CT is an earlier and more sensitive indicator of edema severity across a broader dynamic range (46, 47). Furthermore, the authors developed an automated algorithm to segment the CSF from the CT scans of stroke patients (46) to facilitate and scale up such approaches. However, their method measures changes in the brain volume (BV); thus, it cannot distinguish edema from infarct growth or hemorrhagic transformation. Moreover, it is not suitable for patients with stroke in the brainstem or cerebellum.

#### Net Water Uptake

**Figure 3:**
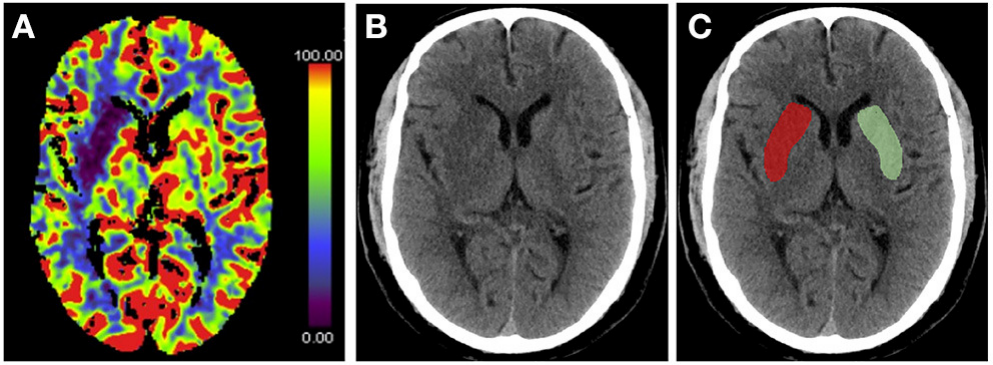
Measurement of % net water uptake (NWU) per volume on admission. **(A)** The initial ischemic core is identified by the initial relative cerebral blood flow (CBF) volume using a threshold of <30% in CBF maps. **(B)** Non-enhanced computed tomography shows a slight decrease in the density of the right basal ganglia region. **(C)** The mean density of the initial core (D_ischemic_) (red) was calculated in relation to the normal density (D_normal_) (green) in the homotopic contralateral region. NWU = 1 – D_Ischemic_/D_Normal_.

Recent studies have proposed the use of NWU within the lesion area to determine the volume of edema (12, 48–50). NWU is calculated using the equation NWU = 1 – D_Ischemic_/D_Normal_, where D_Ischemic_ (Hounsfield Unit, HU) is the density of the ischemic core with hypoattenuation, and D_Normal_ is the density of the same area in the contralateral normal tissue (21, 49, 51) (Figure 3). This quantitative method is based on a physical principle in which the product between the volume of a body and its mean CT density remains constant, regardless of the volume of water uptake (49). Therefore, the increased water content is proportional to the NWU, and the edematous component of the infarct lesion can be quantified using CT densitometry according to the following equation: edema volume = lesion volume × NWU.

CT-based NWU quantification has been described as a precise method to determine the individual volume of edema (21). A previous study demonstrated that it is related to histopathological measurements of the volume of water uptake (52). Fu et al. proposed a new image patch-based NWU procedure that only uses non-enhanced admission CT without the need for lesion segmentation (53). However, this approach has other limitations; for example, patients with pre-existing stroke or significant carotid stenosis may have hypodense lesions, which affect NWU measurement.

### Magnetic Resonance Imaging

Similar to CT, cerebral edema can also be assessed by MLS and change in CSV on MRI because BV increases as edema progresses. Moreover, the intensity characteristics of MRI reflect the tissue composition, and some sequences are particularly sensitive to changes in water content.

#### T2 and T2 Fluid-Attenuated Inversion Recovery

T2 prolongation is commonly observed hours after stroke and is considered to be related to increased water content in ischemic tissue, which represents ionic and vasogenic edema (54, 55). Gerriets et al. found that a significant T2 signal increase is detectable as early as 20–45 min following middle cerebral artery occlusion in rats (56). However, in the early phase of stroke, a slightly increased T2 signal intensity may be masked by high CSF signal. Therefore, T2-weighted fluid-attenuated inversion recovery (FLAIR) sequences can be more sensitive because of the inhibition of CSF signals. In several studies, FLAIR hyperintensity has been measured by calculating the intensity ratio between the stroke lesion and the corresponding normal contralateral hemisphere as a marker to quantify vasogenic edema (57–59).

#### Diffusion Magnetic Resonance Imaging

**Figure 4:**
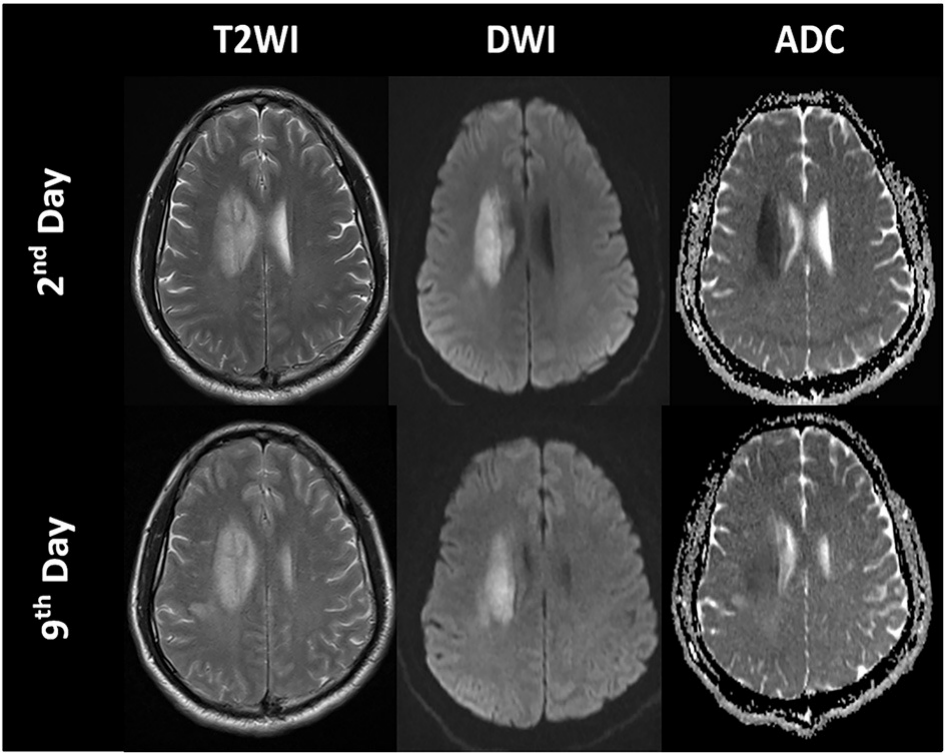
Time course of the signal intensities in T2-weighted imaging (T2WI), diffusion-weighted imaging (DWI), and apparent diffusion coefficient (ADC) maps of a patient with a right paraventricular infarct.

**Figure 5:**
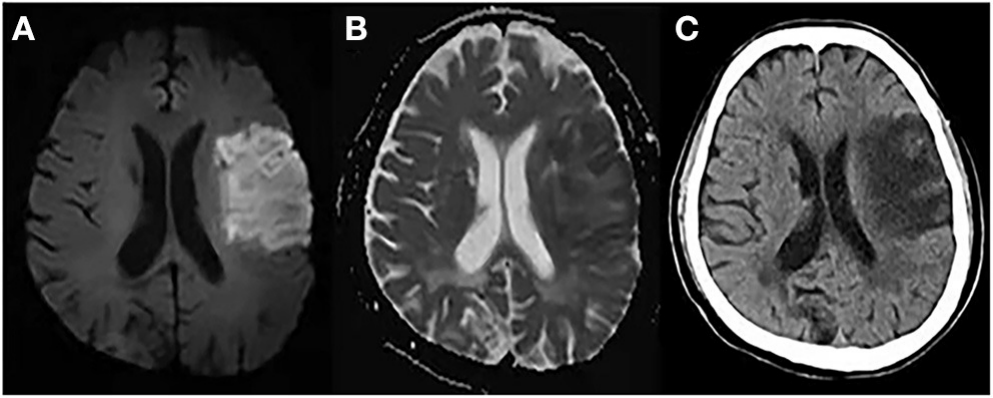
Cytotoxic edema, ionic edema, and vasogenic edema on MRI and CT. A 69-year-old male underwent MRI and CT on day 5 post-stroke. **(A)** DWI shows hyperintensity in the left frontal and parietal lobes. **(B)** ADC map shows hypointensity in the marginal area of the lesion, indicating cytotoxic edema, and hyperintensity in the lesion center, indicating ionic and vasogenic edema. **(C)** CT image shows hypodensity in the left frontal and parietal lobes, especially in the area of ionic and vasogenic edema (i.e., ADC hyperintensity area).

Diffusion MRI is sensitive to the diffusion of water molecules in biological tissue and plays a critical role in the research and clinical management of acute stroke. Diffusion-weighted imaging (DWI) is the most frequently used technique to detect cytotoxic edema. An increase in DWI and a decrease in apparent diffusion coefficient (ADC) can be observed several hours after acute ischemia because of extracellular fluid loss and swelling of various cellular compartments, which are proportionate to the degree of intracellular water accumulation (60, 61). ADC declines immediately when the cerebral blood flow (CBF) falls below 20 to 40 ml/100 g/min in animals and humans (22), and the most dominant decay occurs within the first hours (1–1.5 h) (57, 61, 62). Subsequently, ADC values increase in the days following stroke because of progressive ionic and vasogenic edema and cell lysis, which results in a phenomenon called *ADC pseudo-normalization* (63) (Figures 4, 5). Therefore, timely imaging (24–48 h after stroke onset) is essential to evaluate the process of cytotoxic edema.

However, DWI provides relatively limited quantitative information regarding the microstructural features of brain tissue. Recently, several advanced diffusion techniques have been proposed, such as the ball-and-stick model, q-ball imaging, diffusion spectrum imaging, composite hindered and restricted models of diffusion, and neurite orientation dispersion and density imaging (NODDI) (64–67). Among these, NODDI provides quantification of the relative contribution of the three diffusion compartments (CSF-like, extra-neurite, and intra-neurite) to the total diffusion signal in each voxel (65). NODDI parameters can be used to further elaborate microstructural changes within ischemic tissue and may disentangle the confounding factors behind cerebral edema.

## Neuroimaging Predictors of Edema

The measurement methods of edema have been described above. Because edema strongly influences patient prognoses, predicting its occurrence in the early stages is vital to provide timely intervention. To date, various clinical and imaging markers have been developed to predict edema. In this review, we focus on neuroimaging predictors.

### Infarction Volume

A large ischemic core is considered a key risk factor for ME. Occlusion of both the middle cerebral (MCA) and internal carotid arteries (ICA) is a strong predictor of MLS (11, 18, 68, 69). For patients with MCA occlusion, over 50% of the MCA territory infarction on initial CT predicts ME (18, 70), whereas an acute DWI volume of >80 ml on MRI acquired within 6 h of stroke onset and that of >145 ml when imaged 14 h from stroke onset have been shown to predict rapid early neurological deterioration and the need for neurosurgery (11, 71–74).

A large cerebellar hemisphere infarction is the major cause of severe edema in infra-tentorial infarcts and results in acute obstructive hydrocephalus and potentially fatal tonsillar herniation. Decompressive suboccipital craniectomy with dural expansion with or without ventriculostomy leads to acceptable functional outcomes in most patients. However, cerebellar infarction volume that is predictive of the need for decompression has not yet been established (75, 76).

The early development of a large ischemic area suggests extensive cytotoxic edema. Because this may be associated with a proximal clot, in combination with a poor leptomeningeal collateral status, it can further lead to prominent vasogenic edema.

### Intracranial Volume Reserve

Intracranial volume reserve is represented by the space occupied by the CSF, which is mainly determined by pre-existing brain atrophy. A larger intracranial volume reserve provides space to compensate for the increased BV and can alleviate early neurological deterioration.

The assessment of BV and/or CSV can quantify the degree of brain atrophy (72, 77, 78). The DWI high-intensity volume/BV ratio and cerebral blood volume lesion volume/CSV ratio have been demonstrated as reliable predictive markers for malignant MCA infarction, with a cut-off value of 0.078 (sensitivity 86%, specificity 87%) and 0.92 (sensitivity 96.2%, specificity 96.2%), respectively (72, 79). However, BV information is not readily available to clinicians during the management of acute stroke patients because traditional image processing methods involve sophisticated post-processing steps and are thus relatively slow. However, the application of deep learning-based segmentation methods can provide fast and accurate results (47), which offers great potential for future clinical practice.

Intercaudate distance (ICD) is considered a convenient and practical marker for brain atrophy (80), and a higher ICD has been shown to be a protective factor against malignant infarction (74, 78, 80). Lee et al. demonstrated that an ICD ≥ 20 mm in the non-infarcted hemisphere has an independent protective effect against malignant clinical outcomes during admission to hospital. However, it is also negatively associated with a modified Rankin Scale score of <4 at 6 months (80). These studies indicate that although intracranial volume reserve can compensate for the space-occupying edema during the early phase of stroke, it also represents a pre-existing neurodegenerative process that affects patients' long-term outcomes.

### Blood–Brain Barrier Permeability

The BBB is the interface between blood circulation and brain tissue; it consists of a continuous endothelial membrane within brain microvessels and is sheathed by mural vascular cells and perivascular astrocyte end-feet. During ischemia, several pathological mechanisms, such as inflammation and oxidative stress, can disrupt BBB integrity and increase paracellular permeability, which contributes to vasogenic edema (81, 82).

In clinical studies, the permeability of the BBB (BBBP) is usually measured by the amount of contrast agent that leaks into the extravascular space (83, 84). Hom et al. analyzed 32 patients with acute anterior circulation stroke within 12 h of stroke onset and found that BBBP > 7 ml/100 g/min at admission is 100% sensitive and 79% specific in predicting symptomatic hemorrhagic transformation and ME. Moreover, specificity further increases to 100% after adding age (≥65 years) and tissue plasminogen activator (tPA) administration (81). Compared with cerebral hemorrhage, cerebral edema may have a lesser degree of BBB disruption because it only needs to be permeable to small molecules, such as albumin, rather than blood cells. Unfortunately, the clinical application of BBBP measurement is limited by the use of contrast agents. Recently, a new MRI method based on diffusion-weighted arterial spin labeling (ASL) was proposed as a method to quantify the rate of water exchange across the BBB; it has the advantages of repeatable measurement for longitudinal monitoring and being exempt from the need for a contrast agent (85, 86).

### Collateral Status

Robust pial arterial collaterals may temporarily preserve blood flow during stroke, and collateral status has been shown to be related to post-stroke cerebral edema. Various methods have been proposed to evaluate collateral status based on different imaging models, such as the American Society of Interventional and Therapeutic Neuroradiology or Thrombolysis in Cerebral Infarction grades on digital subtraction angiography (87), as well as the Alberta Stroke Program Early CT Score based on CT angiography (88) or CT perfusion (89). Additionally, new methods, such as ASL, have also been introduced (90).

Collateral status has been demonstrated to be a predictor of cerebral edema in ischemic stroke (91, 92). Jo et al. reported that a collateral status score of <2 strongly predicts malignant cerebral edema [odds ratio (OR): 0.165, 95% confidence interval (CI): 0.064–0.426] (92). Poor collateral status is known to augment the progression of the infarct core and induce more proximal vascular occlusion, which are both associated with brain edema (93, 94).

In patients undergoing recanalization, those with initially poor collaterals may develop greater early brain edema and have a higher early edema progression rate (EPR) (1.6% EPR per one collateral status point) 24 h after acute ischemic stroke (25, 95). This may result in elevated interstitial pressure, increased resistance of collateral arterioles, and downstream perforating arterioles in the hypoperfused area. Subsequently, ischemic edema may be further aggravated, which results in adverse functional outcomes. Huang et al. found that a low collateral score may be an independent risk factor for the development of malignant cerebral edema after mechanical thrombectomy, especially in patients with successful reperfusion (93).

Therefore, collateral status could be used for the early stratification of adjuvant treatment options after successful vessel recanalization, especially anti-edematous treatment.

### Cerebral Veins

Venous changes in the affected hemisphere after acute ischemic stroke may play a crucial role in determining clinical outcomes (94, 96), given that the venous system is responsible for ~70–80% of the cerebral blood volume. Zhang et al. suggested that a lack of superficial middle cerebral vein filling contributes to poor outcomes following thrombolysis and that this indicator predicts edema progression within 24 h in non-reperfusion patients (97, 98). Xia et al. assessed cortical veins (Labbe, sphenoparietal sinus, and the superficial middle cerebral vein) and found that the absence of cortical venous filling is associated with increased brain edema and a higher risk of malignant cerebral edema (OR, 14.68; 95% CI, 4.03–53.45) (94) regardless of whether patients received reperfusion therapy. The likely pathophysiologic mechanism of these signs is the elevation of venous pressure caused by micro-thrombotic occlusion in venules or endothelium swelling after ischemia (99, 100), which may increase fluid leakage into the perivascular space, resulting in brain edema (101).

### Recanalization and Reperfusion

Recanalization therapies in the hyperacute phase following ischemic stroke, such as intravenous thrombolysis with tPA and endovascular thrombectomy, aim to reopen the occluded artery, which has been unequivocally shown to restore CBF in salvageable ischemic tissue and reduce patient disability (102, 103). However, pre-clinical data using rodent and primate models have indicated that tPA facilitates the development of BBB damage in acute ischemic stroke by inducing phasic secretion of matrix metalloproteinase-9, and reperfusion injury is observed after the restoration of vascular supply to ischemic lesions. Both of these conditions may augment the development of edema following recanalization therapy (104, 105). On the contrary, several studies have demonstrated that thrombolytic therapy and recanalization in the hyperacute phase reduce brain edema by arresting infarct growth and rescuing at-risk ischemic tissue (18, 45, 93, 106, 107). Thorén et al. analyzed 22,184 patients who underwent recanalization therapies (intravenous thrombolysis and thrombectomy with or without intravenous thrombolysis) and found that patients who had successful recanalization had a lower cerebral edema grade at 24–36 h than that of those who did not undergo recanalization (13 vs. 23.6%; OR: 0.52) (45). Additionally, *post-hoc* analysis of the Echoplanar Imaging Thrombolytic Evaluation Trial and Mechanical Retrieval and Recanalization of Stroke Clots Using Embolectomy cohorts found that increasing reperfusion is associated with and independently predicts less MLS and a lower swelling volume 3–8 days after stroke onset (107). However, downstream reperfusion is not always achieved even after complete recanalization (called futile recanalization), which is likely related to microvascular obstruction. Nawabi et al. reported that futile recanalization after receiving successful endovascular recanalization (thrombolysis in cerebral infarction scale 2b/3) is associated with an elevated edema volume in the follow-up CT 24 h later (12).

## Artificial Intelligence in the Assessment and Prediction of Edema

Artificial intelligence has been widely used in the segmentation of intracranial tissues. Following the theory that CSF displacement reflects the extent of edema, Chen et al. developed a computer algorithm capable of automatically segmenting CSF from standard clinical CT images to evaluate edema and further refined this algorithm by training a fully convolutional neural network. This new method automatically performs segmentation of clinical CT images with high concordance to manually obtained measurements and takes <1 min per scan (108).

Different types of neural network algorithms have been introduced in the past several decades to predict cerebral edema (109, 110). Compared with traditional regression models, these new methods have higher accuracy. However, studies that investigated these models are often conducted in a single center with relatively small sample sizes. Thus, further research is needed to validate these methods.

## Conclusion

Severe cerebral edema following ischemic stroke is associated with a poor prognosis if timely intervention is not provided. Neuroimaging is important in the assessment of edema and can be used to evaluate the degree of cerebral edema and quantify edema volume. Using different imaging modalities, a range of neuroimaging indicators to predict edema progression have been offered, although their predictive value varies between studies. Therefore, further research is required to establish evaluation and prediction models of cerebral edema and improve their clinical applicability.

## Author Contributions

XZ conceived and drafted the review with guidance from RZ. RZ and PH critically revised the manuscript. All authors carefully reviewed the content and approved the final manuscript version for publication.

## Funding

This study was supported by the National Natural Science Foundation of China (Grant Nos. 81771820 and 82101987), the Natural Science Foundation of Zhejiang Province (Grant Nos. LSZ19H180001 and LQ20H180015), the China Post-doctoral Science Foundation (Grant No. 2019M662083), the Office of China Post-doctoral Council (Grant No. PC2020117), and the Post-doctoral Science Foundation of Zhejiang Province.

## Conflict of Interest

The authors declare that the research was conducted in the absence of any commercial or financial relationships that could be construed as a potential conflict of interest.

## Publisher's Note

All claims expressed in this article are solely those of the authors and do not necessarily represent those of their affiliated organizations, or those of the publisher, the editors and the reviewers. Any product that may be evaluated in this article, or claim that may be made by its manufacturer, is not guaranteed or endorsed by the publisher.
